# *Borrelia miyamotoi* in Human-Biting Ticks, United States, 2013–2019

**DOI:** 10.3201/eid2712.204646

**Published:** 2021-12

**Authors:** Guang Xu, Chu-Yuan Luo, Fumiko Ribbe, Patrick Pearson, Michel Ledizet, Stephen M. Rich

**Affiliations:** University of Massachusetts–Amherst, Amherst, Massachusetts USA (G. Xu, C.-Y. Luo, F. Ribbe, P. Pearson, S.M. Rich);; L2 Diagnostics, New Haven, Connecticut, USA (M. Ledizet)

**Keywords:** *Borrelia miyamotoi*, *Ixodes scapularis*, *Ixodes pacificus*, ticks, bacteria, spirochetes, vector-borne infections, United States

## Abstract

During 2013–2019, *Borrelia miyamotoi* infection was detected in 19 US states. Infection rate was 0.5%–3.2%; of *B. miyamotoi–*positive ticks, 59.09% had concurrent infections. *B. miyamotoi* is homogeneous with 1 genotype from *Ixodes scapularis* ticks in northeastern and midwestern states and 1 from *I. pacificus* in western states.

*Borrelia miyamotoi*, a relapsing fever group spirochete (*1*), was first isolated from *Ixodes persulcatus* ticks in Japan in 1995 (*2*) and later detected in *Ixodes* ticks in the United States and Europe (*3*–*5*). Although *B. miyamotoi* bacteria have been mainly detected in *I. ricinus* species complex ticks that transmit *B. burgdorferi* worldwide, the vector specificity needs further study because investigators have found *B. miyamotoi* in multiple tick species (*6*). *B. miyamotoi* has 3 geographically distinct genotypes: Asian, European, and American. In the United States, *B. miyamotoi* bacteria have been found in field-collected *I. scapularis* ticks in the northeastern and northern midwestern regions, where the average infection rate is 1.9% (*7*). However, an expanded geographic study of the prevalence of *B. miyamotoi* in human-biting ticks, its genotypes, and concurrent infections with other tickborne pathogens is warranted.

Human-biting ticks were submitted to the public tick testing program at the University of Massachusetts (Amherst, Massachusetts, USA) during May 2013–December 2019. We extracted DNA from individual ticks using the Epicenter Master Complete DNA and RNA Purification Kits (Lucigen, https://www.lucigen.com). We performed a species-specific quantitative PCR (qPCR) for differentiation of *I. scapularis* and *I. pacificus* ticks (*8*). To detect *Borrelia* bacteria, we first applied a genus-specific detection assay, followed by specific qPCR assays for *B. burgdorferi* sensu lato and *B. miyamotoi*. We detected the tickborne pathogens *Anaplasma phagocytophilum*, *Babesia microti*, *B. mayonii,* and *Ehrlichia muris*–like agent (EMLA) by a multiplex qPCR assay targeting different genes. We used a qPCR assay targeting tick 16S mtDNA gene as an internal control (*8*). We sequenced 3 partial gene fragments, 16S rDNA (*16S*) (*9*), flagellin (*fla*) (*6*), and glycerophosphodiester phosphodiesterase (*glpQ*) (*6*), for *B. miyamotoi* samples that were positive by qPCR.

We received and tested 39,198 ticks found on humans for *B. miyamotoi* during May 2013–December 2019. Of those, 38,855 (99.12%) ticks originated from the continental United States, comprising 18 tick species (Table). Although *Ixodes* ticks are the main vectors for *B. miyamotoi*, we did not detect *B. miyamotoi* DNA in *I. affinis*, *I. angustus*, *I. cookei*, *I. dentatus*, *I. marxi*. *I. muris,* or *I. spinipalpis* ticks. We detected *B. miyamotoi* in *I. pacificus* (14/1,497, 0.94%) and *I. scapularis* (594/34,621, 1.72%) ticks.

**Table:** Human-biting tick species positive for *Borrelia miyamotoi* and *B. burgdorferi* sensu lato*,* United States, 2013–2019

Tick species	Total no. tested	No. *B. miyamotoi* positive	No*. B. burgdorferi* s.l. positive
*Amblyomma americanum*	1,167	0	0
*A. * *cajennense*	1	0	0
*A. * *maculatum*	8	0	0
*Dermacentor andersoni*	60	0	0
*D. occidentalis*	91	0	0
*D. variabilis*	1,060	0	0
*Haemaphysalis leporispalustris*	2	0	0
*H. longicornis*	7	0	0
*Ixodes affinis*	2	0	0
*I. angustus*	55	0	0
*I. cookei*	123	0	0
*I. dentatus*	48	0	7
*I. marxi*	26	0	0
*I. muris*	9	0	2
*I. pacificus*	1,497	14	25
*I. scapularis*	34,621	594	11,287
*I. spinipalpis*	63	0	3
*Rhipicephalus sanguineus*	15	0	0
Total	38,855	608	11.324

*B. miyamotoi* was found in 19 states; infection rates were 0.5%–3.2% (Figure). In the western United States, *B. miyamotoi* was found in *I. pacificus* ticks in Oregon and California (14/1,497, 0.94%). Although *I. scapularis* ticks are distributed across the eastern United States, no *B. miyamotoi*–positive ticks were detected south of Virginia. *B. miyamotoi*–positive ticks were concentrated in the Northeast and upper Midwest (594 of 34,621, 1.72%) (Figure). Lyme disease remains the principal public health concern; the causative agent, *B. burgdorferi* (11,287/34,621; 32.60%, 95% CI 32.1%–33.1%), was 19 times more prevalent than *B. miyamotoi* (594/34,621, 1.72%) in *I. scapularis* ticks.

**Figure:**
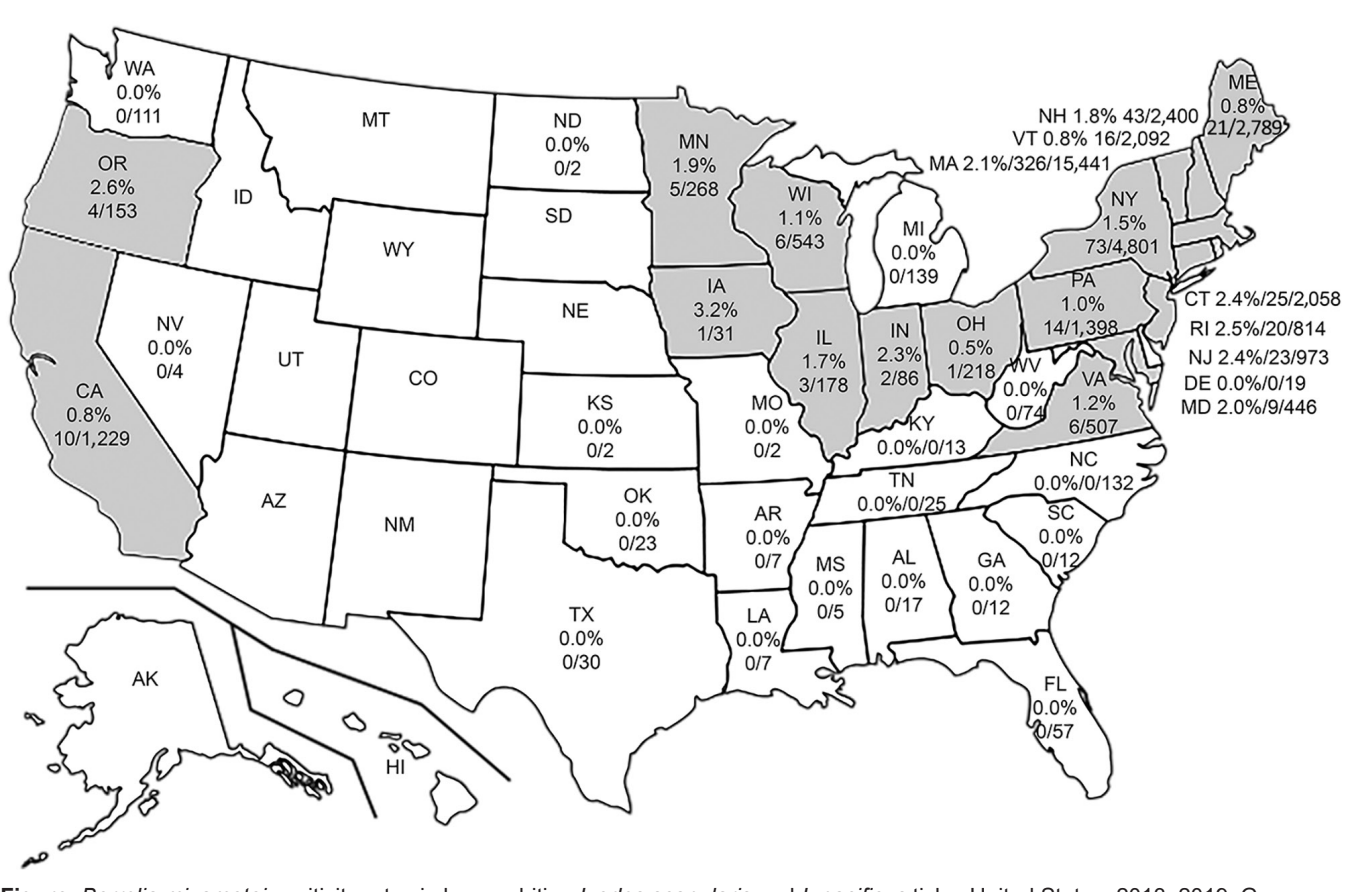
*Borrelia miyamotoi* positivity rates in human-biting *Ixodes scapularis* and *I. pacificus* ticks, United States, 2013–2019. Gray shading indicates states in which *B.miyamotoi* was detected in human-biting ticks.

On average, prevalence of *B. miyamotoi* infection in *I. scapularis* ticks (1.72%, 95% CI 1.58%–1.86%) was higher than in *I. pacificus* ticks (0.94%, 95% CI 0.51%–1.56%). The prevalence of *B. miyamotoi* in *I. pacificus* ticks was 1.00% (95% CI 0.53%–1.7%) in adults (13/1,300), 0.53% (95% CI 0.01%–2.9%) in nymphs (1/190), and 0.00% (95% CI 0%–40.1%) in larvae (0/7). The prevalence of *B. miyamotoi* in *I. scapularis* ticks was 1.80% (95% CI 1.64%–1.97%) in adults (456/25,376), 1.54% (95% CI 1.29−1.83%) in nymphs (133/8,615), and 0.79% (95% CI 0.26%–1.84%) in larvae (5/630).

Of 594 *B. miyamotoi*–positive *I. scapularis* ticks, 351 (59.09%) had concurrent infections. We found 293 (49.33%) *I. scapularis* ticks had a dual infection with *B. miyamotoi*: 220 (37.04%) were also infected with *B. burgdorferi* s.l., 43 (7.24%) with *A. phagocytophilum*, and 30 (5.05%) with *B. microti*. We further found 52 (8.75%) had a triple infection with *B. miyamotoi*: 23 (3.87%) were also infected with *B. burgdorferi* s.l. and *A. phagocytophilum*, 22 (3.70%) with *B. burgdorferi* s.l. and *B. microti*, and 7 (1.18%) with *A. phagocytophilum* and *B. microti*. Six (1.01%) of the *B. miyamotoi*–positive ticks had a quadruple infection with *B. miyamotoi*, *B. burgdorferi* s.l., *A. phagocytophilum*, and *B. microti*. No ticks with *B. mayonii* or EMLA were additionally infected with *B. miyamotoi*.

Multilocus sequence typing of the *16S*, *fla*, and *glpQ* genes revealed 2 distinct *B. miyamotoi* genotypes separated by their tick vectors, *I. scapularis* ticks in the Northeast and upper Midwest and *I. pacificus* ticks in the West (Appendix). Whereas the *16S* gene sequences were identical among all isolates, variable sites were found among *fla* and *glpQ* nucleotide sequences. Among 14 *I. pacificus* tick–borne *B. miyamotoi* isolates, all *fla* and *glpQ* sequences were identical. A previously reported A/G substitution in *B. miyamotoi fla* sequences from *I. pacificus* ticks (*5*,*9*) was outside of our sequenced *fla* fragment (Appendix). The genetic identity between the 2 tick species–specific genotypes was 0.996 for *fla* and 0.986 for *glpQ*. Unlike heterogeneous *B. burgdorferi* populations, *B. miyamotoi* appears to be very homogeneous within its respective tick vectors.

Appendix Additional information about *Borrelia miyamotoi* in human-biting ticks, United States, 2013–2019.
