# Supplementary material for: Borrelia miyamotoi in Human-Biting Ticks, United States, 2013–2019
Source: Emerg Infect Dis. 2021 Dec;27(12):3193–5. doi: 10.3201/eid2712.204646 (PMC8632181; doi:10.3201/eid2712.204646)
Supplement: Appendix — Additional information about Borrelia miyamotoi in human-biting ticks, United States, 2013–2019. [file 20-4646-Techapp-s1.pdf]

Article DOI: https://doi.org/10.3201/eid2712.204646

Borrelia miyamotoi in Human-Biting Ticks, United States, 2013–2019

Appendix

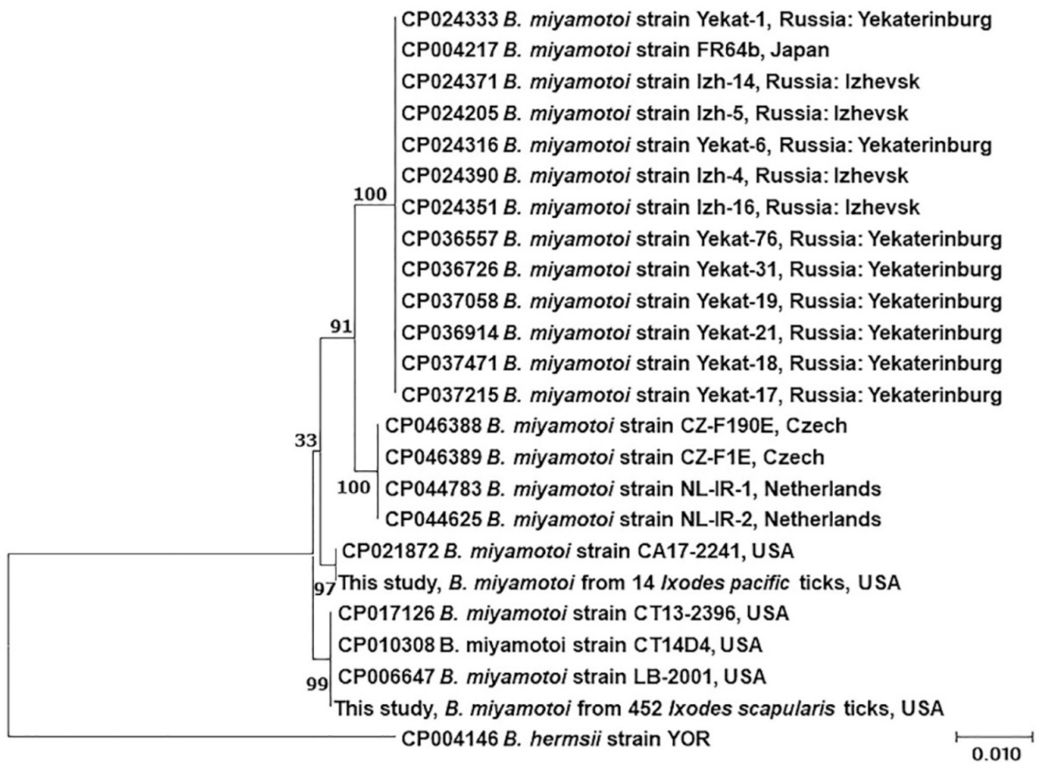

Appendix Figure 1. Phylogenetic tree of *Borrelia miyamotoi* 16S rDNA (*16S*), flagellin (*fla*), and glycerophosphodiester phosphodiesterase (*glpQ*) genes constructed by maximum likelihood method of MEGA software version 10 (http://www.megasoftware.net). Of 594 *B. miyamotoi*–positive *Ixodes scapularis* ticks, we successfully sequenced a 1,545bp long fragment of 3 concatenated genes from 452 ticks. We selected Hasegawa-Kishino-Yano with invariable site as the best model based on Bayesian information criterion scores. Numbers on the branches represent bootstrap support with 500 bootstrap replicates. Scale bar represents nucleotide substitutions per site.

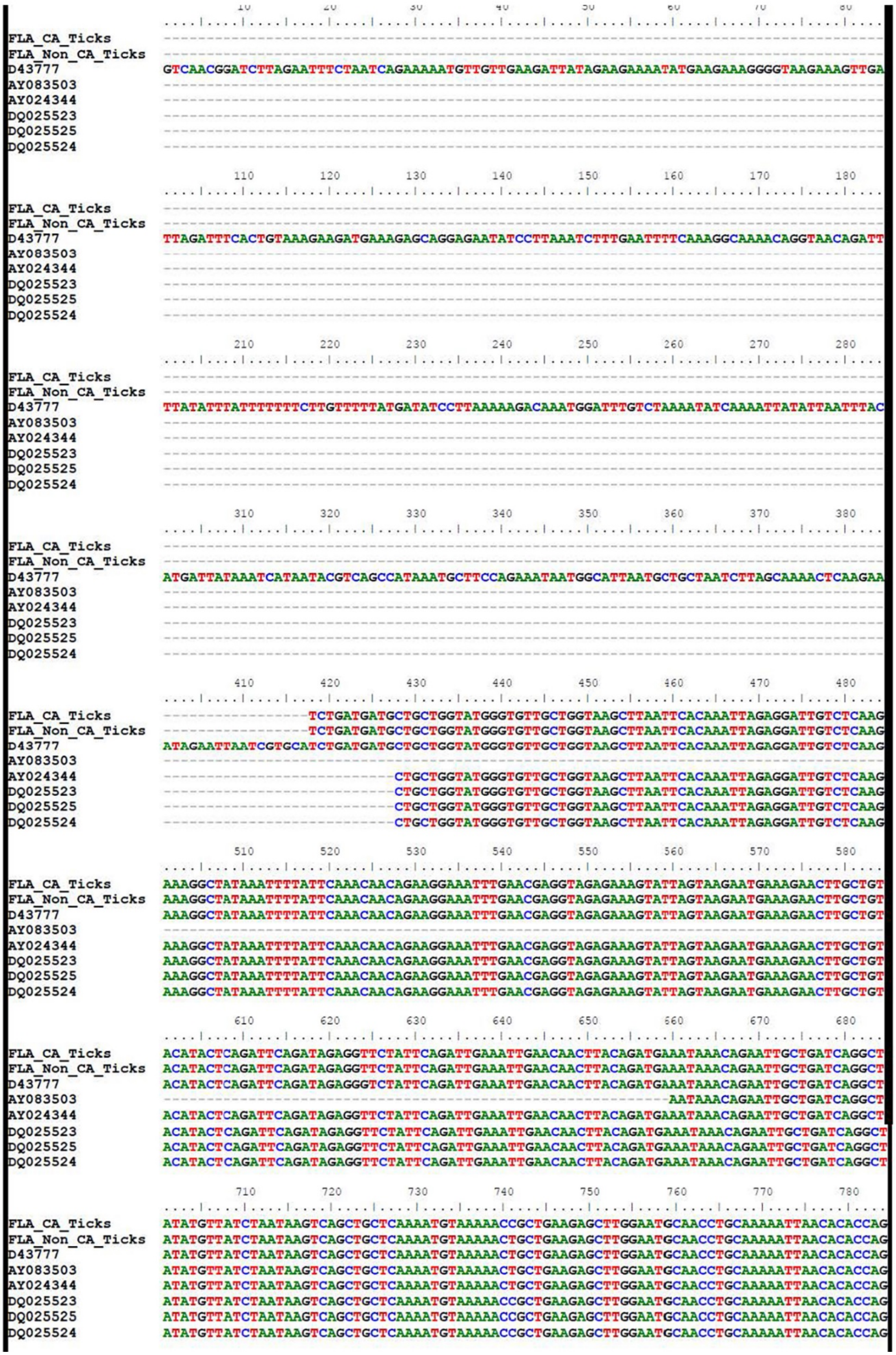

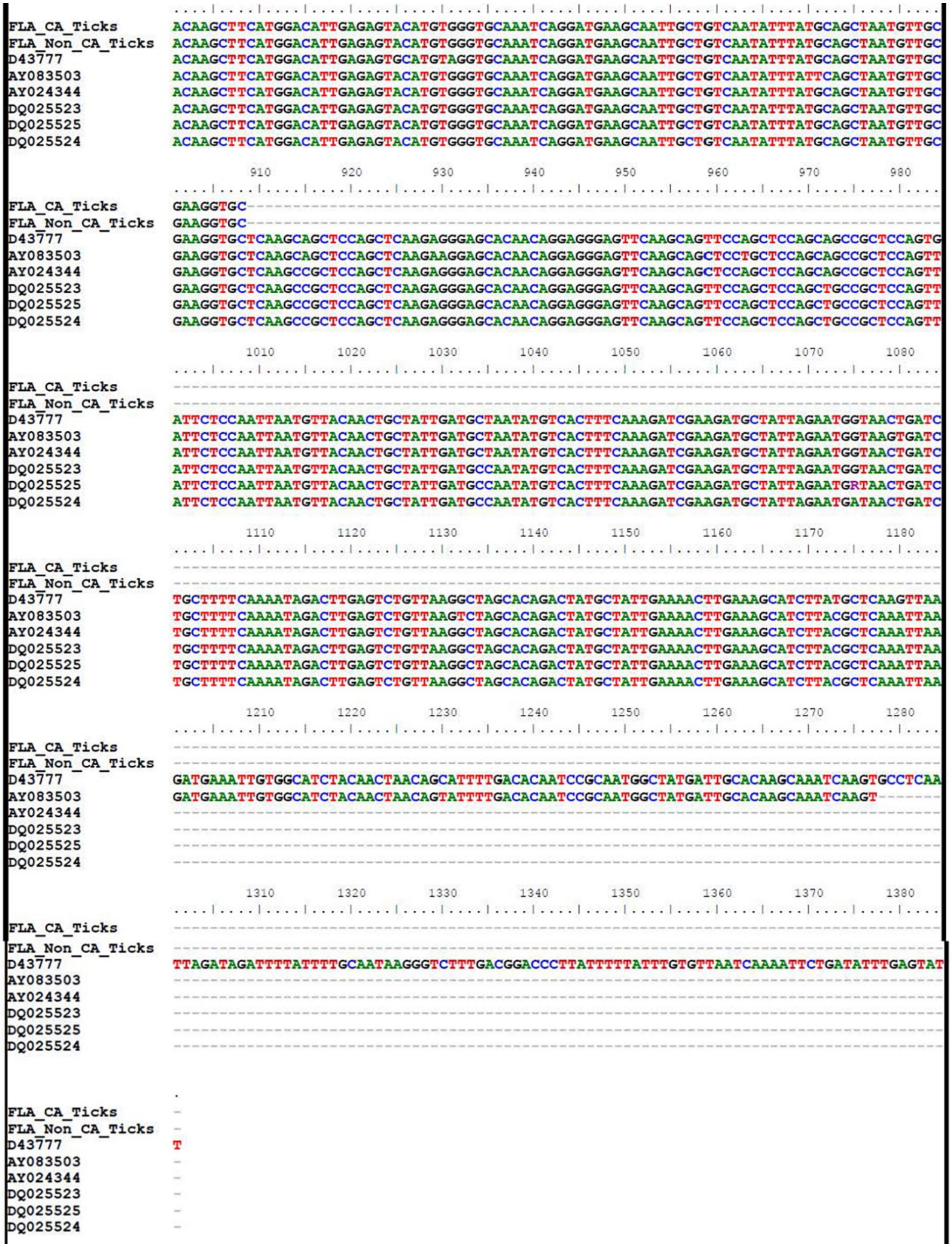

Appendix Figure 2. Alignment of *Borrelia miyamotoi fla* gene segment.
